# Transition to innovative, human-relevant pre-clinical cardiovascular research: a perspective

**DOI:** 10.1093/cvr/cvae080

**Published:** 2024-03-08

**Authors:** Evangelos P Daskalopoulos, Pierre Deceuninck, Maurice Whelan, Laura Gribaldo

**Affiliations:** European Commission, Joint Research Centre (JRC), Via E. Fermi 2749, 21027 Ispra (VA), Italy; European Commission, Joint Research Centre (JRC), Via E. Fermi 2749, 21027 Ispra (VA), Italy; European Commission, Joint Research Centre (JRC), Via E. Fermi 2749, 21027 Ispra (VA), Italy; European Commission, Joint Research Centre (JRC), Via E. Fermi 2749, 21027 Ispra (VA), Italy

**Keywords:** Cardiovascular diseases, Biomedical research, Non-animal methods, New approach methodologies, Innovation

Cardiovascular diseases (CVDs) remain the leading cause of mortality and morbidity worldwide. It is estimated that approximately 17.9 million people died from CVDs in 2019, which represented 32% of all deaths globally. Coronary heart disease is the commonest type of CVDs, contributing to the death of more than 380 000 people in 2020 in the USA,^1^ while other pathologies—including stroke, arrhythmias, heart valve diseases, cardiomyopathies, aneurysms, heart failure, and congenital defects—are also very debilitating. According to recent data, the EU expenditure for CVDs is ∼210 billion EUR per year, while the annual direct and indirect CVD-related costs in the USA were recently estimated at 407.3 billion USD.^1^ It is evident therefore that CVDs constitute a huge societal problem and a colossal burden for healthcare systems.

Existing therapies (interventional or pharmacological) for CVDs largely aim to treat symptoms and slow down the progression of a cardiovascular pathology rather than to deliver a cure. Furthermore, the situation is being exacerbated by lifestyle factors common in western societies including smoking and alcohol use, lack of exercise, and unhealthy diets. Clearly therefore, more action is needed on several fronts. In response, the European Commission (EC) recently conveyed its determination to reduce the burden of non-communicable diseases (NCDs)—including CVDs—by its ‘Healthier Together Initiative’, as well as by endorsing international partnerships such as the ‘European Alliance for Cardiovascular Health’ (EACH) and by actively supporting research and innovation through Horizon Europe (2021-27) under Cluster 1 (Health).

Experimental investigation has been instrumental in biomedical research to shed light on disease mechanisms and to develop our diagnostic and therapeutic arsenal. Thus far, animal models have contributed substantially to our understanding of the pathophysiology and molecular mechanisms implicated in the development of various CVDs and to the translation of findings on promising treatments from the lab to the clinic.^2^ Although animal models may partially mimic some human cardiac (patho)physiological features, they fail to fully recapitulate all aspects and complexity of human physiology and CVDs. For example, functional parameters such as heart rate differ enormously between humans and the most commonly used rodent models (i.e. mice and rats). Furthermore, rodent heart architecture, cell distribution and functionality, and the expression of several cardiac genes under physiological and pathological conditions are very different in comparison to humans. These inherent differences can considerably limit the interpretation and translation of responses observed in animal species and represent a key factor underpinning the major problem of drug attrition. It is characteristic that 9 in 10 of all new drug programmes fail to reach market authorization. Notably, the failure rate in the cardiovascular therapeutic group is one of the highest.^3^ There are many reasons for these high attrition rates linked to poor predictivity of human responses including lack of efficacy, unpredicted side effects, and the early (and possibly unnecessary) termination of a drug candidate due to misleading indications of toxicity in the development process. It is estimated that only 17% of the positive (for cardiotoxicity) cases found in rodents are actually confirmed as positive in humans!^4^

In the endeavour to reduce the impact of translational failures, research efforts are gradually shifting towards bridging the gap between animal models (pre-clinical phase) and humans (clinical phases) by adopting more human-relevant and predictive approaches. Research strategies for the development of safe and efficacious therapies against CVDs are beginning to focus on innovative non-animal methods—based on complex *in vitro* and *in silico* models for example—that better recapitulate human (patho)physiology and functionality. To aid this transition, the EU Reference Laboratory for alternatives to animal testing (EURL ECVAM), part of the EC’s Joint Research Centre (JRC), undertook a unique study to examine emerging new approach methodologies (NAMs) being used for CVD research. As a result, Celi *et al.*^5^ recently published a systematic review on *Advanced Non-animal Models in Biomedical Research – Cardiovascular Diseases*. This includes a technical report that describes the review methodology and presents the main findings, accompanied by a carefully curated data catalogue. Both are free to download from the EURL ECVAM collection in the JRC data catalogue.

The study was based on a systematic review of peer-reviewed scientific publications spanning from 2013 to 2019, which identified and evaluated more than 14 700 abstracts describing CVD-relevant NAMs. The review eventually selected a total of 449 NAMs used for CVD research, according to carefully defined criteria. These NAMs mainly use computer modelling and simulation (*in silico*), cell or tissue cultures (*in vitro*), and cells or tissues explanted from a living organism (*ex vivo*).

The review highlighted *in silico* approaches as the most prevalent category of non-animal models used in CVD research. These integrate mathematics, biophysics, biomechanics, computer science, biology, and even electrophysiology, as well as imaging data (MRI, ultrasound, or CT), to simulate a given cardiovascular function and pathology. Such innovative *in silico* methods are increasingly used in the last years and are expected to revolutionize biomedicine and translation to the clinic, by providing vital data for target identification, early stage drug discovery, and drug repurposing. The ‘digital twin’^6^ concept—gaining increasing interest—is a virtual tool based on an individual’s characteristics (e.g. genetics, lifestyle, and physiological parameters). This concept involves the continuous integration of clinical data (e.g. laboratory results, physiological, and imaging data), statistical analyses, *in silico* simulations, and mechanistic knowledge to capture the uniqueness of individuals and is expected to accelerate the vision of personalized medicine. It is worth noting that Passini *et al*.^7^ demonstrated that *in silico* trials perform much better than animal approaches in predicting cardiotoxicity associated with pro-arrhythmia, suggesting that computational simulations could even be incorporated into frameworks for the safety risk assessment in the not so distant future.

*In vitro* approaches were the second most abundant category identified by the review. These can be subdivided into *in vitro* models with cells [e.g. 2D/3D cultures, organoids, and organ-on-chip (OoC)] and *in vitro* models without cells (e.g. aortic grafts, decellularized valve patches, and 3D-printed models). Although advanced *in vitro* models were largely under-represented in the review, it is evident that such approaches—e.g. engineered heart tissue, spheroids, living tissue slices, OoC, and organoids—are emerging as promising approaches for use in biomedical research and early pre-clinical drug development.^8^ Possibly the most rapidly evolving approaches are based on OoC, which are complex engineered microfluidic systems comprising 2D or 3D cell cultures. The technology already exists to employ heart-on-chip to model a wide range of cardiovascular pathologies, such as inherited cardiomyopathies, cardiac fibrosis, or ischaemia–reperfusion injury, and to conduct drug compound screening and cardiotoxicity studies, while similar vessel-on-chip approaches are also being developed to study inflammation, thrombosis, etc.^9^ There is now clear evidence on how OoC approaches can accurately recapitulate complex human cardiovascular (patho)physiology and can change the way we conduct pre-clinical CVD research and drug development. Notably, OoC technologies have the potential to transform the way we approach drug research and development (R&D), and it is estimated that the wider use of OoC can potentially reduce costs for the R&D of a new drug compound by 10–26%.^10^

The 2021/2784(RSP) Resolution adopted by the European Parliament (EP) in September 2021 urged for a coordinated EU-wide action plan to phase out animal use for scientific and regulatory purposes ‘as soon as scientifically possible and without lowering the level of protection for human health and the environment’. Although the Resolution acknowledged the valuable contribution of animal models in progressing our understanding of human disease over the years, it specifically highlighted the importance of innovative approaches (e.g. sophisticated computer simulations, OoC, and other complex *in vitro* approaches) and stressed that the acceleration of the transition towards animal-free methods and technologies will be essential for any tangible change to occur. Nearly a year after the EP Resolution, the *FDA Modernization Act 2.0* was unanimously approved by the US Senate to eliminate an 84-year-old requirement that all investigational medicines should first be tested on animals, before being used in human clinical trials. Thus, it is evident that the political will to accelerate the transition to innovative research methodologies is strong on both sides of the Atlantic. What is still missing, however, is a wider realization from the scientific community (including the cardiovascular field) that more efforts are required to move forward at a faster pace. Disruptive innovative technologies like *in silico* approaches and OoC—in combination with machine learning, artificial intelligence, and high-performance computing—have the potential to transform the way we conduct basic and applied research, develop new drug therapies, and ultimately treat patients. Gaps and challenges exist however (*Table 1*) that need to be addressed. It is the right time for the scientific community to act, in order to challenge mind sets, to push for more innovation, and to pave the way for doing better, more predictive, and more human-relevant cardiovascular research, to exploit more human-relevant methods and reach the ultimate goal of personalized medicine in CVDs.

**Table 1:** Advantages and challenges of the most commonly used NAMs in cardiovascular biomedical research

	Advantages	Challenges
*In silico*	Fast	Standardization is lagging behind
	Reliable/more accurate vs. animal methods in predicting cardiotoxicity/making prediction of the ability of a compound to bind to a receptor even before compound synthesis	Can be closed systems—transparency is an issue/wider use is prevented Conditions of access to patient data for training/using models are not regulated yet
	Can provide mechanistic insights into special high-risk populations for CVDs Expected to reduce the cost for R&D by ∼35% Decrease/prevent altogether the use of animals	Complex models may require access to large computing facilities (not part of standard healthcare facilities)
	Can combine with AI/ML and high throughput to deliver huge amounts of data in less time	
	Can integrate a wide range of data from different disciplines, towards the digital twin	
OoC	Ease of use—user-friendly and portability	Standardization is lagging; validation/qualification of OoC models is also missing
	Fast	Compatibility with other lab equipment, imaging, analytical instrumentation, etc. can be an issue
	Fabrication of chips is quite cheap	Integration of sensors might be problematic in some cases
	Expected to reduce the cost for drug R&D by ∼10–26%	Adsorption effects on the surface of OoC, which can affect the results (use of alternative materials for the manufacturing of the chip)
	Decrease/prevent altogether the use of animals	3D cultures are hard to maintain long term
	Integration of sensors can measure in real-time several parameters (pH, O_2_, etc.)	Human-derived cardiac cells (e.g. stem cells or primary cell lines) are not mature enough/variability of maturation protocols
	Good mimicking of the cardiovascular (myocardial/vessel) microenvironment	Need to acquire human-derived cells from several donors
	iPSC-derived CMs and CFs can be produced	Mostly ventricle derived (atrial-derived models are under-represented)
	Can obtain iPSCs from donors with specific background (age, sex, race/ethnicity, and pathologies) to study specific CVDs	Chronic aspect of development of CVDs is challenging to reproduce
	Possible to test a variety of drug compounds in a wide range of doses at the same time	
	Ability to integrate several chips together (multi-chips), e.g. heart-on-chip with liver-on-chip and/or kidney-on-chip, to better mimic the human physiology	

AI/ML, artificial intelligence/machine learning; CFs, cardiac fibroblasts; CMs, cardiomyocytes; CVDs, cardiovascular diseases; iPSC, induced pluripotent stem cells; NAMs, new approach methodologies; OoC, organ-on-chip; R&D, research and development.

## Data Availability

No new data were generated or analysed in support of this research.

## Authors

**Biography:** Evangelos P. Daskalopoulos (Vangelis) studied pharmacy in the UK, and following a year of working as an NHS hospital pharmacist, he returned to his native Greece to conduct a PhD thesis on basic pharmacology (drug metabolism). After acquiring his doctorate, he moved in the field of cardiovascular research, studying pharmacological targets (Maastricht University, The Netherlands) and pathophysiological mechanisms (UCLouvain, Belgium) of post-MI cardiac remodelling. After 15 years of working in academia, he was intrigued to learn more about the roles the European Commission plays in making sense of knowledge to inform policymaking in Europe. To this end, he joined the Joint Research Centre (JRC) in Ispra and worked for 2 years in the EURL ECVAM, supporting knowledge-sharing activities towards the promotion of new approach methodologies (NAMs) in research. Since January 2023, Vangelis is working as a Policy Officer in DG RTD in Brussels, contributing to the implementation of the European research and innovation (R&I) policy that is related to chemicals and advanced materials and following relevant files on NAMs, FAIR data, etc.

**Biography:** Pierre Deceuninck serves as a data scientist within the Systems Toxicology Unit at the European Commission’s Joint Research Centre (JRC). He plays an active role in the annual production of statistical reports pertaining to the use of animals for scientific research purposes. His responsibilities involve the compilation and analysis of data provided by member states of the European Union, all within the framework of Directive 2010/63/EU. Pierre is presently leading the ‘Innovation and Impact of Biomedical Research’ project. The primary goal of this project is to enhance the influence of innovation in the realm of biomedical research. His main goals include identifying and promoting new approach methodologies, bridging gaps between research communities, improving research methodologies, and fostering cross-disciplinary collaboration.

**Biography:** Prof Maurice Whelan is Head of the Systems Toxicology Unit of the Directorate for Health and Food of the European Commission’s Joint Research Centre (JRC), based in Ispra, Italy. He also heads the JRC’s EU Reference Laboratory for alternatives to animal testing (EURL ECVAM). Maurice is the EU co-chair of the OECD Advisory Group on Molecular Screening and Toxicogenomics that is responsible for the OECD programme on Adverse Outcome Pathways; a member of the Steering Committee of the European Partnership for Alternative Approaches to Animal Testing (EPAA); and chair of the newly created Regulatory Advisory Board of the European Organ-on-Chip Society (EUROoCS). His publications include over 200 scientific papers and a recent book on the validation of alternative methods for toxicity testing. He has held a number of external appointments including the 2017–18 Francqui Chair for alternative methods at the Vrije Universiteit Brussel (VUB, Belgium) and is currently a visiting Professor of Bioengineering at the University of Liverpool (UK).

**Biography:** Laura Gribaldo, MD, PhD in microbiology and virology, has years of experience in the field of testing for safety assessment. She set up and managed a transcriptomic platform for the development of standardized assays in toxicology, and she was responsible to support a programme ensuring harmonization and validation of procedures in genetic testing for diagnostics purposes. For the Public Health Unit, she worked on Rare Diseases, representing JRC at the EUCERD meetings for the establishment of the European platform for rare diseases registry. Until the end of July, she was responsible for the studies on non-animal models in biomedical research at the System Toxicology Unit, where she still works in the field of knowledge sharing, dissemination, education, and training as Active Senior.
